# Red dichromatic imaging improves visibility of bleeding during gastric endoscopic submucosal dissection

**DOI:** 10.1038/s41598-023-35564-z

**Published:** 2023-05-26

**Authors:** Kohei Oka, Naoto Iwai, Takashi Okuda, Toshifumi Tsuji, Hiroaki Sakai, Chie Hattori, Masashi Taniguchi, Tasuku Hara, Toshiyuki Komaki, Junichi Sakagami, Keizo Kagawa, Osamu Dohi, Yoshito Itoh

**Affiliations:** 1 Department of Gastroenterology and Hepatology, Fukuchiyama City Hospital, 231 Atsunakamachi, Fukuchiyama-City, Kyoto, 620-8505 Japan; 2 Department of Molecular Gastroenterology and Hepatology, Graduate School of Medical Science, Kyoto Prefectural University of Medicine, Kyoto, Japan

**Keywords:** Oesophagogastroscopy, Upper gastrointestinal bleeding

## Abstract

Bleeding frequently occurs during gastric endoscopic submucosal dissection (ESD) and bleeding points are sometimes difficult to detect. Red dichromatic imaging (RDI) was recently developed to improve the visibility of bleeding. Our study aimed at examining the efficacy of RDI in improving the visibility of bleeding during gastric ESD. We retrospectively evaluated the visibility score and color difference of bleeding spot during gastric ESD during September 2020–January 2021. The visibility score was evaluated as four numeric values by operators, and the color difference between the bleeding spot and surroundings was evaluated using RDI and white light imaging (WLI). A further analysis to evaluate bleeding characteristics was performed to evaluate the possible beneficial effects of RDI. Twenty patients with a total of 85 bleedings were analyzed. The mean visibility score in RDI was significantly higher than that in WLI (3.69 ± 0.60 vs 3.20 ± 0.84, *p* < 0.01). The color difference with RDI was also significantly higher than that with WLI (19.51 ± 15.18 vs 14.80 ± 7.41, *p* < 0.01). Furthermore, in the bleedings with a higher visibility score in RDI, the color difference in RDI was significantly higher than that in WLI (23.99 ± 19.29 vs 14.33 ± 7.08, *p* < 0.01). The multivariate analysis of visibility scores revealed that submergence of bleeding points was independently associated with the superiority of RDI (odds ratio 10.35, 95% confidence interval: 2.76–38.81, *p* < 0.01). Our study demonstrates that RDI can improve the visibility of bleeding during gastric ESD.

## Introduction

Endoscopic submucosal dissection (ESD) is widely accepted as a minimally invasive therapy for early gastric cancer^1,2^. Unfortunately, bleeding is reported as a major complication of gastric ESD^3,4^. Severe hemorrhages that complicate identification of bleeding points are occasionally encountered. Implementing hemostasis using hemostatic forceps is a known method to prevent bleeding during ESD procedure^5,6^; however, excessive or frequent coagulation can cause carbonation in the submucosal layer, consequently resulting in disturbance during gastric ESD^7^. Improving visibility of bleeding may contribute to quality-controlled ESD by preventing excessive or frequent hemostasis.

Red dichromatic imaging (RDI), formerly referred to as dual red imaging, is a novel image-enhanced endoscopy that is characterized using red and amber wavelengths (600 nm and 630 nm)^8^. Previous studies have evaluated its benefit in terms of bleeding visibility using prototype endoscopes during gastric ESD^9–11^. RDI is anticipated to enhance the color contrast between the bleeding points and surrounding pooled blood. A few reports have examined the usability of RDI in the treatment of esophageal varices or acute gastrointestinal bleeding using objective measures such as color difference^12,13^. However, it remains unestablished how RDI enhances contrast in bleeding during gastric ESD. In addition, direct evidence of the efficacy of RDI on identifying bleeding points during gastric ESD is limited.

Therefore, our study evaluated the efficacy of RDI for bleeding during gastric ESD, using both objective and subjective measurements.

## Methods

### Study design

In this retrospective study, we reviewed the data of 31 consecutive patients who underwent gastric ESD at Fukuchiyama City Hospital from September 2020 to January 2021. The inclusion criterion consisted of the presence of coagulation for intraoperative bleeding with a shift from white light imaging (WLI) to RDI. The visibility of bleeding during ESD was compared between WLI and RDI. For this purpose, the visibility score of the bleeding point as a subjective measure was defined by the operators and the color value and difference between the bleeding points and surrounding pooled blood were calculated as objective measures. In addition, the visibility score was analyzed between experienced (≥100 cases of ESD) and non-experienced (<100 cases of ESD) operators. Furthermore, an analysis of the color difference according to whether RDI could improve the visibility score or not, was performed to elucidate the association between the visibility score and color difference. Finally, a sub-analysis of the visibility score to evaluate the beneficial effect of RDI in terms of the bleeding characteristics, was performed. The study was conducted in accordance with the Declaration of Helsinki, and the protocol was approved by the Institutional Review Board of Fukuchiyama City Hospital (No. 2–47). The opt-out method was used to obtain informed consent owing to its retrospective design.

### Visibility score analysis

The operators evaluated visibility score immediately after ESD. We defined the visibility score as four numeric values according to previous studies where 1 corresponds to poor visibility and almost impossible to detect and 4 corresponds to excellent visibility and easy to detect. Moreover, numeric value 2 refers to fair visibility and slightly possible to detect with careful observation, whereas numeric value 3 refers to good visibility and possibility of detection with careful observation^14,15^.

### Color difference analysis

We calculated the color difference according to the procedure given in the CIE 1976 L^*^a^*^b color space (CIELAB). The CIELAB defines a three-dimensional color system that represents human color perception where L^*^ represents lightness, a^*^ represents the red/green axis, and b^*^ indicates the yellow/blue axis^16^. Three points at 3 × 3 pixels each in the bleeding points and in the surrounding pooled blood were plotted as the regions of interest (ROIs) in the images that were extracted from videos and had similar compositions in WLI and RDI. The flood selection tools of the image editing software Affinity Photo 1.10.4 (Serif. Inc, Nottingham, UK) were applied to select pixels of similar color and CIELAB color values in ROIs were obtained. We compared the averages of L^*^, a^*^, and b^*^ color values in ROIs between the bleeding points and surrounding pooled blood and calculated the color value differences between WLI and RDI as ΔL, Δa, and Δb. Finally, we calculated the color difference or ΔE, which represent the distance between the bleeding points and surrounding pooled blood on the three-dimensional color space CIELAB, using the following formula:

$$\Delta {\text{E }} = \left[ {\left( {\Delta L} \right)^{2} + \left( {\Delta a} \right)^{2} + \left( {\Delta b} \right)^{2} } \right]^{1/2}$$

**Figure 1:**
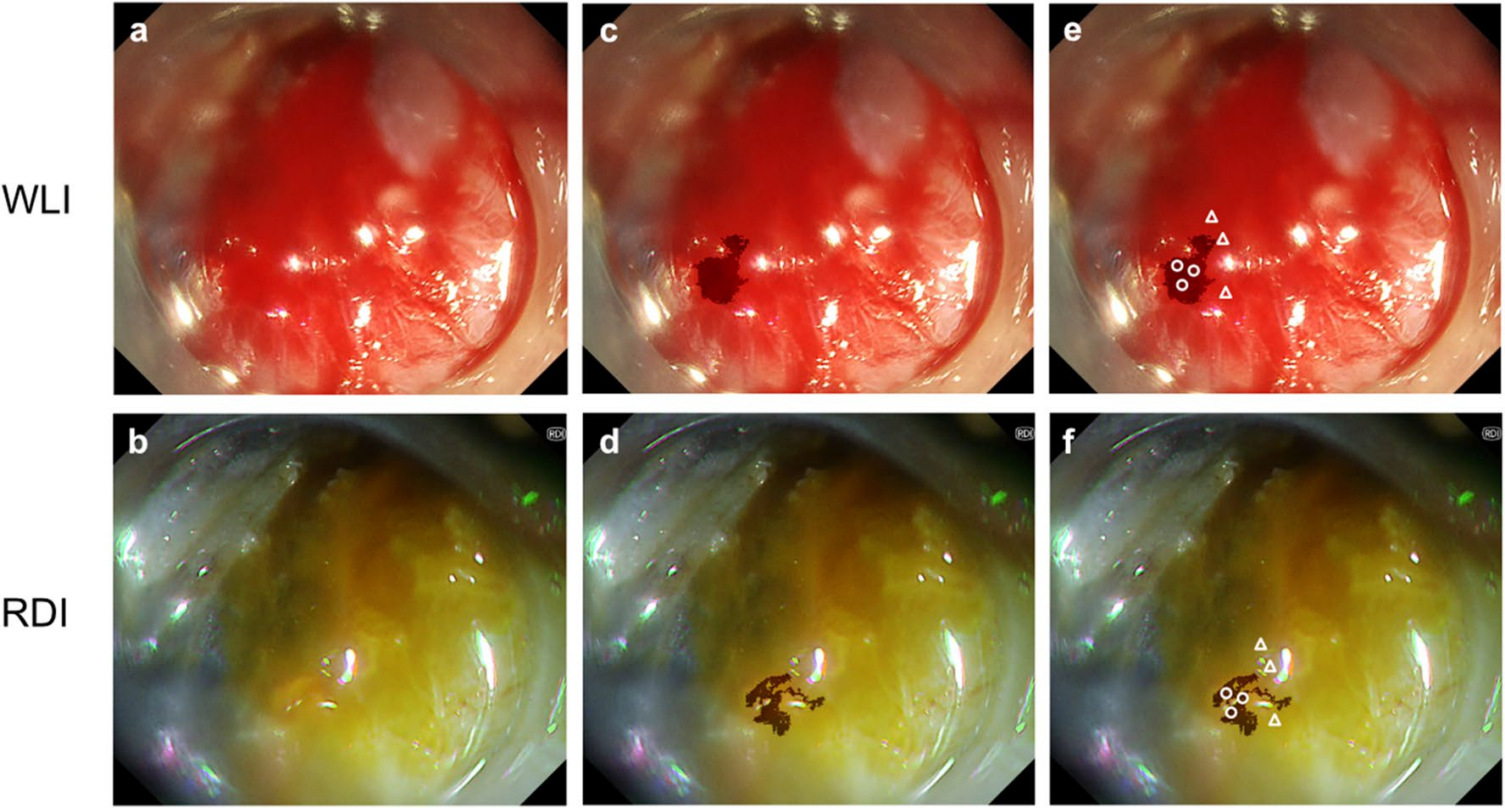
(**a**, **b**) Representative images of the bleeding during gastric endoscopic submucosal dissection on white light imaging (WLI) and red dichromatic imaging (RDI). (**c**, **d**) The dark color area represents the bleeding point that is determined by selecting pixels of a similar color. (**e**, **f**) The white circles indicating region of interest (ROI) in the bleeding point were selected in the area, and the white triangles indicating ROI in the surrounding pooled blood were selected from outside of the area.

**Figure 2:**
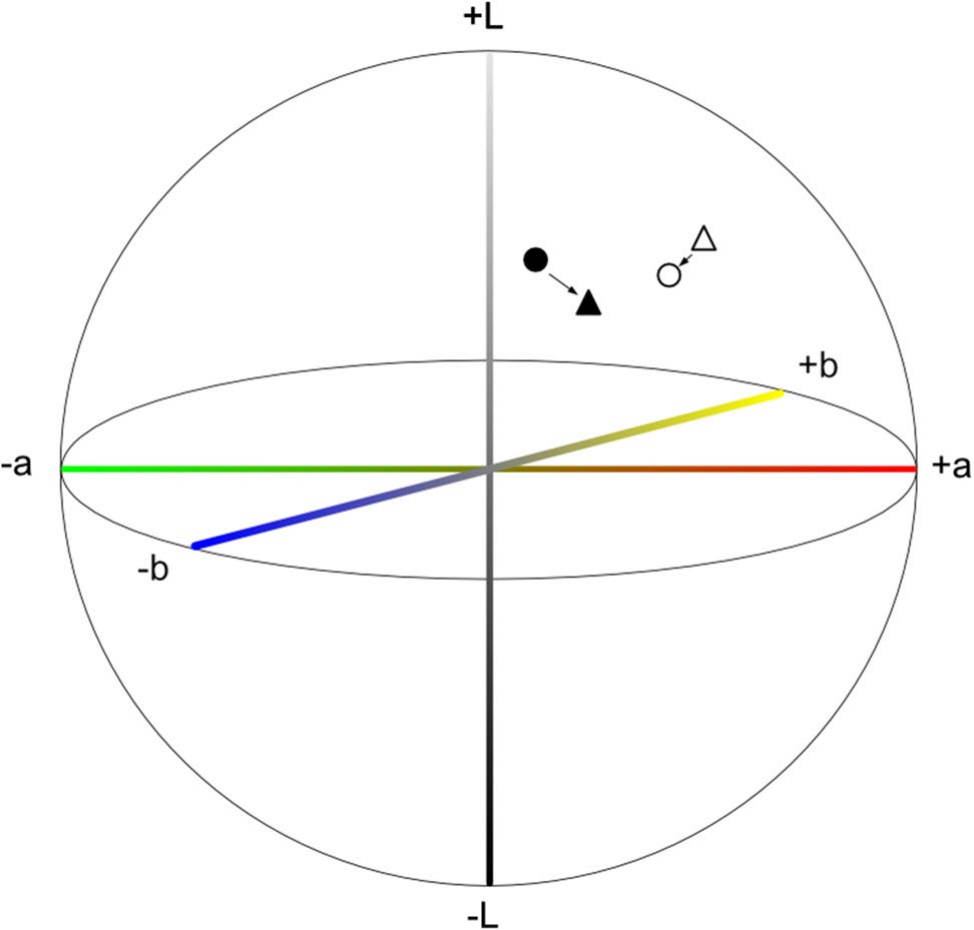
The CIE 1976 L^*^a^*^b color space expressed on three-dimensional geometry with the bleeding point plotted. The outlined circle and triangle indicate the color values on white light imaging (WLI). The filled circle and triangle indicate the color values on red dichromatic imaging (RDI).

Figure 1 shows the representative images of bleeding and the selection of the ROI. Figure 2 shows a representative color difference on the CIELAB.

### Evaluation of bleeding characteristics with RDI

**Figure 3:**
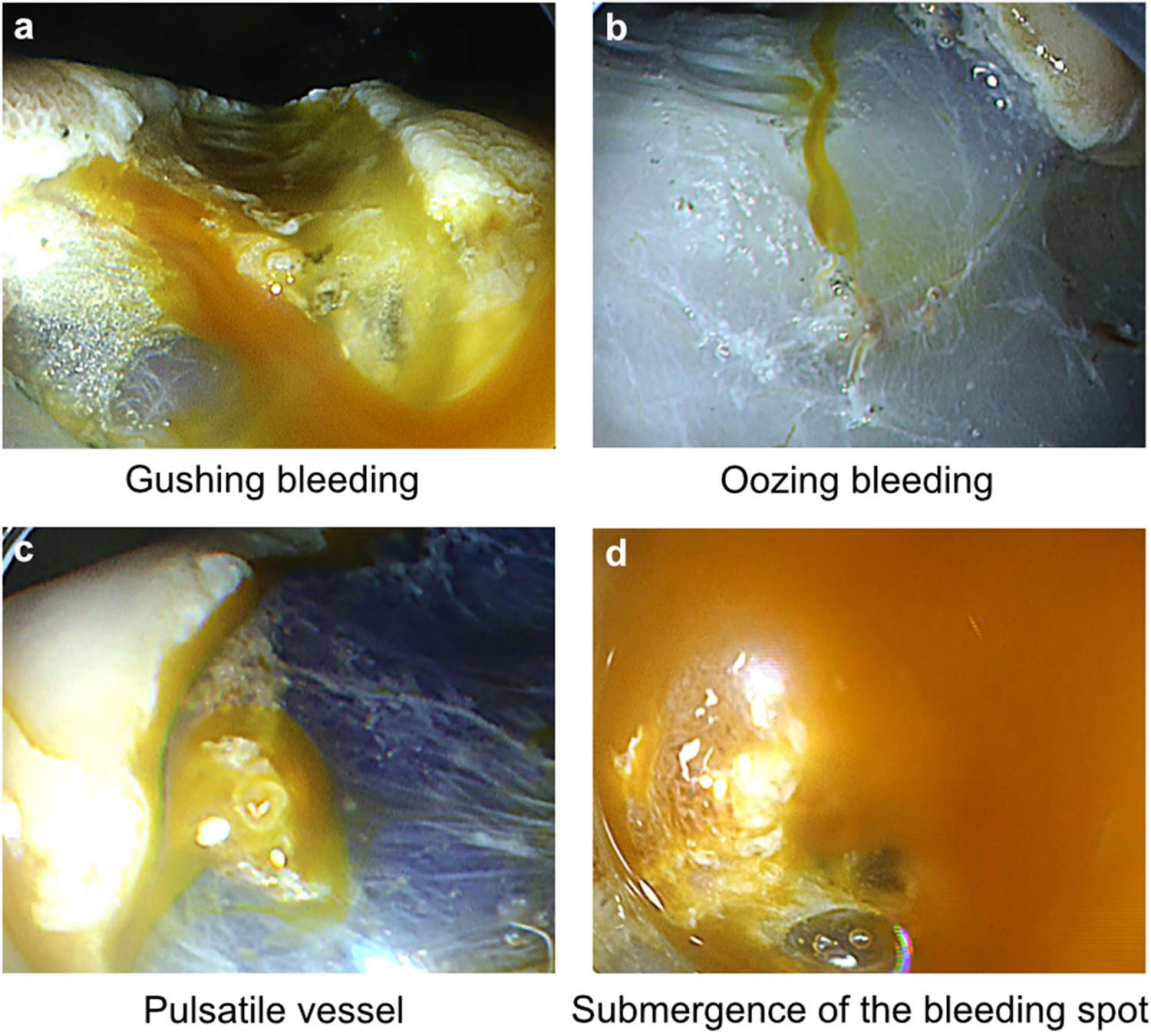
Representative images of bleeding categories on RDI. (**a**) Gushing bleeding, (**b**) oozing bleeding, (**c**) presence of pulsatile vessel, and (**d**) submergence of the bleeding spot. RDI, red dichromatic imaging

We evaluated three categories of bleeding visibility using the captured video images: bleeding type (gushing or oozing), presence of a pulsatile vessel (present or absent), and submergence of the bleeding point (present or absent). The representative images of these categories are shown in Fig. 3. The classifications were performed by a single endoscopist (K.O.). We then assessed the improvement of the bleeding characteristics with RDI using the visibility score.

### Endoscopic submucosal dissection

A mucosal incision and submucosal dissection were performed. The bleeding point was evaluated using WLI followed by RDI when hemostasis was required for bleeding. All coagulation procedures were performed only on RDI. The time for hemostasis and the number of coagulations attempted were calculated.

All ESD procedures were performed using the EVIS X1 endoscopic system with a GIF-H290T endoscope, which were installed at our institution in August 2020. We used ITknife2, DualKnife, or a 3.5 mm long Clutch Cutter (Fujifilm Medical, Tokyo, Japan). RDI mode 1 was used that was preferred for hemostasis^17^. Submucosal injections with a mixture of 0.6% sodium alginate solution Liftal K, (Kaigen Pharma Co., Tokyo, Japan), physiological saline solution, and indigo carmine were administered. Endoscopic hemostasis was performed using ITknife2, Clutch Cutter, or Coagraspar G with a VIO 3 electrosurgical unit (Erbe Elektromedizin GmbH, Tübingen, Germany). The procedures were recorded using a high-definition medical video recorder UR-4MD (TEAC Europe GmbH, Wiesbaden, Germany). All devices were produced by Olympus Co. Tokyo, Japan unless stated otherwise. The procedures were performed by five operators (K.O., N.I., T.O., T.T., and H.S.) among whom three (N.I., T.O., and T.T.) were experienced operators (≥100 of ESD) and two (K.O. and H.S.) were non-experienced operators (<100 cases).

### Statistical analysis

Numerical variables are presented as mean ± standard deviation. Comparisons between the two groups were performed using the Wilcoxon signed-rank test. Multivariate logistic regression analysis was performed to evaluate bleeding characteristics with RDI for variables that reached *p* < 0.05 in the univariate analysis. Statistical significance was set at *p* < 0.05. All statistical analyses were conducted using JMP14.3 (SAS Institute Inc., Cary, NC, USA).

## Results

Table 1 summarizes the clinicopathological features of the patients. Eleven patients who did not need hemostasis were excluded. As a result, 85 bleedings in 20 patients were analyzed. The mean age was 66.9 ± 9.8 years. Eighteen patients underwent ESD using ITknife2, one using DualKnife, and one using Clutch Cutter. Among the 85 coagulation cases, 41 hemostasis were performed using ITknife2, 12 using Clutch Cutter, and 32 using Coagraspar G. The mean time for hemostasis and number of coagulations were 66.7 ± 89.0 s and 1.9 ± 1.3, respectively.

**Table 1:** Clinicopathological features.

Clinicopathological features	
No. of patients/hemostasis	20/85
Age, years	66.9 ± 9.8
Gender (men/women), n	17/3
Tumor size, mm	15.2 ± 9.7
Resection size, mm	34.3 ± 6.9
Tumor location, n	
Upper stomach	4
Middle stomach	6
Lower stomach	10
Use of anticoagulant, n	3
Device in ESD procedure, n	
ITknife2	18
Clutch Cutter	1
DualKnife	1
Device in hemostasis, n	
ITknife2	41
Clutch Cutter	12
Coagraspar G	32
Change of hemostasis device, n	13
Time for hemostasis, seconds	66.7 ± 89.0
No. of coagulations, n	1.9 ± 1.3

Data are represented as mean ± standard deviation; *ESD* Endoscopic submucosal dissection.

Table 2 shows the visibility score, color values, and color differences between RDI and WLI. The mean visibility score in RDI was significantly higher than that in WLI (3.69 ± 0.60 vs 3.20 ± 0.84, *p* < 0.01). Among the 85 bleedings, 40 bleedings had a higher visibility score in RDI than in WLI, 45 bleedings had equivalent visibility score between RDI and WLI, and none had a lower visibility score in RDI. In terms of the color values, the lightness (ΔL) in the RDI group was significantly higher than that in the WLI group (13.85 ± 14.27 vs 6.34 ± 5.63, *p* < 0.01), and the red/green axis (Δa) in the RDI group was significantly lower than in the WLI group (−4.76 ± 8.45 vs 8.52 ± 5.93, *p* < 0.01). The color difference (ΔE) in RDI was significantly higher than that in WLI (19.51 ± 15.18 vs 14.80 ± 7.41, *p* < 0.01)

**Table 2:** The visibility score, color values, and color differences between RDI and WLI.

	RDI	WLI	*p* value
Visibility score	3.69 ± 0.60	3.20 ± 0.84	< 0.01
Color values			
Lightness (ΔL)	13.85 ± 14.27	6.34 ± 5.63	< 0.01
Red/green axis (Δa)	−4.76 ± 8.45	8.52 ± 5.93	< 0.01
Yellow/blue axis (Δb)	8.57 ± 7.02	8.01 ± 5.53	0.53
Color difference (ΔE)	19.51 ± 15.18	14.80 ± 7.41	< 0.01

Data are represented as mean ± standard deviation

*RDI* Red dichromatic imaging, *WLI* White light imaging.

Table 3 shows the visibility score according to the operator’s experience related to ESD. Among the 85 coagulation cases, the non-experienced operators performed 50 coagulation cases, whereas the experienced operators performed 35 cases. The visibility scores in RDI were significantly higher than those in WLI in both experienced and non-experienced operators (*p* < 0.01 and *p* < 0.01, respectively)

**Table 3:** The visibility score according to the operator’s experience on ESD.

Operator	No. of bleedings	RDI	WLI	*p* value
Experienced (n=3)	35	3.60±0.12	3.34±0.14	<0.01
Non-experienced (n=2)	50	3.76±0.07	3.10±0.12	<0.01

Data are represented as mean ± standard deviation

*ESD* Endoscopic submucosal dissection, *RDI* Red dichromatic imaging, *WLI* White light imaging.

**Figure 4:**
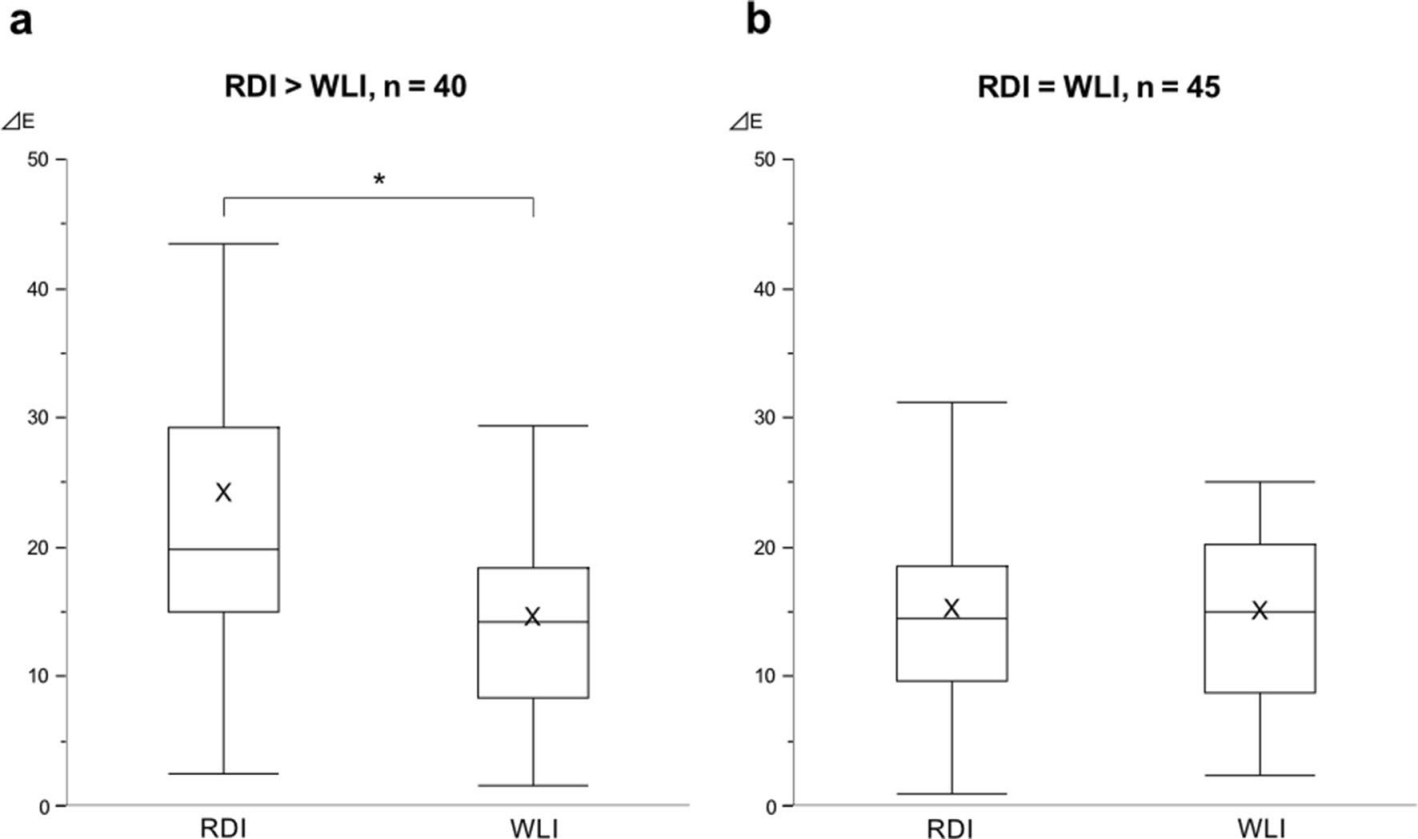
(**a**) Box plot showing color differences (ΔE) with RDI and WLI in bleedings with a higher visibility score in RDI than in WLI. *p < 0.01. (**b**) Box plot showing ΔE with RDI and WLI in bleedings presenting with the equivalent visibility score. The middle line represents the median. The symbol “x” in the box plot represents the mean. ΔE, color difference; RDI, red dichromatic imaging; WLI, white light imaging

Figure 4 displays the color difference between RDI and WLI according to whether RDI could improve the visibility score or not. In the bleeding cases with a higher visibility score in RDI than in WLI (n = 40), the color difference in RDI was significantly higher than that in WLI (23.99 ± 19.29 vs 14.33 ± 7.08, *p* < 0.01). In contrast, in the bleedings with equivalent visibility scores (n = 45), no significant differences in color difference were observed between RDI and WLI (15.53 ± 8.70 vs 15.21 ± 7.74, *p* = 0.43)

Table 4 shows the analysis of bleeding characteristics using the visibility scores of RDI and WLI. Univariate analysis demonstrated that gushing bleeding, the presence of pulsatile vessels, and submergence of the bleeding point were associated with the visibility score (odds ratio [OR] 5.47, 95% confidence interval [CI]: 2.07–14.51, *p* < 0.01; OR 4.67, 95% CI: 1.18–18.41, *p* < 0.05; OR 15.37, 95% CI: 4.60–51.35, *p* < 0.01; respectively). Multivariate analysis showed that submergence of the bleeding point was an independent factor for the superiority of RDI in terms of the visibility score (OR 10.35, 95% CI: 2.76–38.81, *p* < 0.01)

**Table 4:** Analysis of bleeding characteristics using the visibility scores of RDI and WLI.

	Visibility score		Univariate analysis			Multivariate analysis		
	RDI > WLI (n=40)	RDI = WLI (n=45)	OR	95%CI	*p* value	OR	95%CI	*p* value
**Bleeding type**								
Gushing	32	19	5.47	2.07–14.51	<0.01	2.32	0.74–7.26	0.15
Oozing	8	26	1			1		
**Pulsatile vessel**								
Present	10	3	4.67	1.18–18.41	<0.05	1.16	0.21–6.28	0.87
Absent	30	42	1			1		
**Submergence**								
Present	24	4	15.37	4.60–51.35	<0.01	10.35	2.76–38.81	<0.01
Absent	16	41	1			1		

*OR* Odds ratio, *CI* Confidence interval, *RDI* Red dichromatic imaging, *WLI* White light imaging.

## Discussion

Our study demonstrates that RDI can improve the visibility score and color difference associated with bleeding during gastric ESD. We have shown that RDI enhances lightness and decreases redness of the bleeding points, contributing factors to visibility improvement. Additionally, we have shown that RDI improved the visibility score regardless of the operator’s experience related to ESD. Further, we have demonstrated in the visibility score analysis that RDI may be useful where submergence of bleeding points occurs. To the best of our knowledge, this is the first study to evaluate the utility of RDI using both objective and subjective measure, for improving the visibility of bleeding during gastric ESD.

RDI utilizes two long wavelengths: amber light with strong hemoglobin absorption and red light with weak hemoglobin absorption. RDI has been previously reported to enhance the color contrast of bleeding points because the difference in hemoglobin absorption can emphasize blood gradations^11^. A previous study demonstrated that RDI can be used for the detection of bleeding points during gastric ESD using a visibility scale as a subjective measure^9^. Another study reported that RDI can reduce eye movement and psychological stress during ESD^10^, although its efficacy on hemostasis time is controversial^10,11^.

In our study, we observed that 47.1% (40/85) of the bleedings had a higher visibility score in RDI than in WLI, 52.9% (45/85) had an equivalent visibility score, whereas no bleedings had a lower visibility score in RDI. A previous study reported that 55% of bleeding cases during gastric ESD were associated improved visibility in RDI, and 45% had equivalent visibility between RDI and WLI^9^. These subjective findings suggest that approximately half of bleedings would benefit from RDI when hemostasis is needed during gastric ESD. Additionally, the visibility score was consistently better in RDI than in WLI regardless of the operator’s experience, suggesting that even non-familiar operators could benefit from RDI.

Furthermore, we showed that RDI increased the color difference between the bleeding points and surroundings through lightness upregulation and redness suppression. Previous studies have similarly reported that RDI improved color difference in esophageal varices and acute gastrointestinal bleeding^12,13^. We also used CIELAB color values to evaluate the color differences as mentioned in previous reports. In addition, we revealed that the color differences in RDI were higher than those in WLI for bleedings with a higher visibility score in RDI, whereas no differences were observed in the bleedings presenting with equivalent visibility scores. Collectively, our findings suggest that RDI has the potential to provide clear visibility through high-contrast color difference in bleeding during gastric ESD.

Through analysis of the visibility score, we showed that cases with gushing bleeding, presence of pulsatile vessels, and submergence of the bleeding point could benefit from RDI. Unlike a previous study^9^, our results indicated that gushing bleeding may be a desirable target for RDI as it is possible to identify bleeding points under dynamic flow in RDI. In addition, multivariate analysis revealed that RDI could aid in the submergence of bleeding points. Nevertheless, submergence of bleeding points is a challenge as surroundings with pooled blood often present a weak color contrast, thus, disrupting hemostasis. The previous study also reported that RDI improved the visibility of blood pooling during gastric ESD^9^. We speculate that bleeding visibility may be affected by dynamic changes in blood flow as well as color differences in still images. Therefore, further research is needed to establish an assessment method for dynamic changes.

Our study has some limitations. First, this was a retrospective, single-center study with a relatively small sample size. Furthermore, we could not perform a direct comparison of the utility of RDI with that of WLI because all coagulation procedures were performed using RDI. Therefore, a selection bias cannot be excluded. Second, the participant operators had limited experience i.e., fewer than 500 cases. Therefore, our results cannot be applied to expert operators. Third, we performed a color difference analysis using CIELAB color values, although the color values were evaluated only for still images. Another assessment method should be established to objectively evaluate dynamic changes in blood flow.

In conclusion, our study revealed that RDI improved the visibility of bleeding during gastric ESD based on the visibility score and color difference. Furthermore, RDI has the potential to provide clear visibility, especially in submergence of bleeding points.

## Data Availability

The datasets used and/or analyzed during the current study available from the corresponding author on reasonable request.

## References

[1] Gotoda T, Yamamoto H, Soetikno RM (2006). Endoscopic submucosal dissection of early gastric cancer. J. Gastroenterol..

[2] Ono H (2021). Guidelines for endoscopic submucosal dissection and endoscopic mucosal resection for early gastric cancer (second edition). Dig. Endosc..

[3] Jeon SW (2009). Predictors of immediate bleeding during endoscopic submucosal dissection in gastric lesions. Surg. Endosc..

[4] Toyonaga T, Nishino E, Hirooka T, Ueda C, Noda K (2006). Intraoperative bleeding in endoscopic submucosal dissection in the stomach and strategy for prevention and treatment. Dig. Endosc..

[5] Muraki Y (2012). Management of bleeding and artificial gastric ulcers associated with endoscopic submucosal dissection. World J. Gastrointest. Endosc..

[6] Tanaka S (2017). Efficacy of a new hemostatic forceps during gastric endoscopic submucosal dissection: A prospective randomized controlled trial. J. Gastroenterol. Hepatol..

[7] Toyonaga T, Nishino E, Man IM, East JE, Azuma T (2012). Principles of quality controlled endoscopic submucosal dissection with appropriate dissection level and high quality resected specimen. Clin Endosc..

[8] Yahagi N (2019). Dual red imaging: A novel endoscopic imaging technology visualizing thick blood vessels in the gastrointestinal wall. Endosc. Int. Open..

[9] Yorita N (2020). Clinical usefulness of dual red imaging in gastric endoscopic submucosal dissection: A pilot study. Clin. Endosc..

[10] Maehata T (2020). Efficacy of a new image-enhancement technique for achieving hemostasis in endoscopic submucosal dissection. Gastrointest. Endosc..

[11] Fujimoto A (2022). Clinical usefulness of red dichromatic imaging in hemostatic treatment during endoscopic submucosal dissection: First report from a multicenter, open-label, randomized controlled trial. Dig. Endosc..

[12] Furuichi Y, Abe M, Takeuchi H, Yoshimasu Y, Itoi T (2022). Red dichromatic imaging reduces endoscopic treatment time of esophageal varices by increasing bleeding point visibility (with video). Dig. Endosc..

[13] Hirai Y (2022). Evaluation of the visibility of bleeding points using red dichromatic imaging in endoscopic hemostasis for acute GI bleeding (with video). Gastrointest. Endosc..

[14] Yoshida N (2015). Improvement in the visibility of colorectal polyps by using blue laser imaging (with video). Gastrointest. Endosc..

[15] Matsumura S (2021). Improved visibility of early gastric cancer after successful helicobacter pylori eradication with image-enhanced endoscopy: A multi-institutional study using video clips. J. Clin. Med..

[16] Kuehni RG (1976). Color-tolerance data and the tentative CIE 1976 L a b formula. J. Opt. Soc. Am..

[17] Kita A, Tanaka H, Ramberan H, Kuribayashi S, Uraoka T (2021). Endoscopic submucosal dissection of early-stage rectal cancer using full-time red dichromatic imaging to minimize and avoid significant bleeding. VideoGIE..

